# Macular: a multi-scale simulation platform for the retina and the primary visual system

**DOI:** 10.3389/fninf.2025.1726374

**Published:** 2026-02-02

**Authors:** Bruno Cessac, Erwan Demairy, Jérôme Emonet, Evgenia Kartsaki, Thibaud Kloczko, Côme Le Breton, Nicolas Niclausse, Selma Souihel, Jean-Luc Szpyrka, Julien Wintz

**Affiliations:** 1 Université Côte d'Azur, Biovision Team and Neuromod Institute, Inria, Sophia Antipolis, France; 2 Service d'Expérimentation et Développement, Université Côte d'Azur, Inria, Sophia Antipolis, France; 3 P16 - Programme IA, Inria Rocquencourt, Domaine de Voluceau, Le Chesnay-Rocquencourt, France

**Keywords:** graphical user interface, *in silico* experiments, numerical simulations, primary visual system, retina

## Abstract

We developed Macular, a simulation platform with a graphical interface, designed to produce *in silico* experiment scenarios for the retina and the primary visual system. A scenario involves generating a three-dimensional structure with interconnected layers, each layer corresponding to a type of “cell” in the retina or visual cortex. The cells can correspond to neurons or more complex structures (such as cortical columns). Inputs are arbitrary videos. The user can use the cells and synapses provided with the software or create their own using a graphical interface where they enter the constituent equations in text format (e.g., LaTeX). They also create the three-dimensional structure via the graphical interface. Macular then *automatically* generates and compiles the C++ code and generates the simulation interface. This allows the user to view the input video and the three-dimensional structure in layers. It also allows the user to select cells and synapses in each layer and view the activity of their state variables. Finally, the user can adjust the phenomenological parameters of the cells or synapses via the interface. We provide several example scenarios, corresponding to published articles, including an example of a retino-cortical model. Macular was designed for neurobiologists and modelers, specialists in the primary visual system, who want to test hypotheses *in silico* without the need for programming. By design, this tool allows simulation of natural or altered conditions (e.g., pharmacology, pathology, and development).

## 1 Introduction

Our visual system has an extraordinary capacity. It is capable of converting the flow of photons emitted by our environment into a flow of electrical impulses that our brain and consciousness can interpret. This allows us to react quickly and effectively to the movements and changes that are constantly occurring around us. The process starts in the retina. This organ owes its efficiency, on the one hand, to its layered structure, composed of different types of neural layers—from photoreceptors to ganglion cells—connected by specific synapses to form neural circuits that respond to local visual characteristics. On the other hand, the retina is a fundamentally dynamic object. Just as much as its structure, the variety of time scales involved in neural and synaptic processes is essential for enabling the retina to encode visual information efficiently, given that our environment is constantly in motion.

Our knowledge of the retina is essentially based on experimentation. For over a century, this has enabled us to characterize its structure and how a multitude of specific circuits work together to generate a reliable representation of visual scenes. However, given the high level of complexity, spanning a wide range of time scales, experimentation alone cannot provide a holistic description. Furthermore, experiments are costly in terms of resources, time, and energy. In this context, numerical simulation combined with modeling is a valuable asset. Even though no simulation is currently capable of reproducing the behavior of a complete retina, they can reproduce the behavior of a particular circuit or combination of circuits, explore hypotheses, and vary physiological parameters that are difficult to access experimentally. It is therefore natural that numerous retinal simulation platforms have been developed (see Section 7 for a non-exhaustive list).

While the simulation platform we present here, Macular, fits this perspective, it nevertheless differs significantly from existing platforms. Furthermore, although it includes the VirtualRetina simulator developed by members or former members of our group (Wohrer and Kornprobst, 2009), it differs from it in several ways. Macular was designed with several requirements in mind. First, it is intended for experimenters or modelers with no programming knowledge who would like to simulate situations that interest them. Macular offers an interface that allows them to enter equations (e.g., in LaTeX) that characterize the dynamics of specific neurons or synapses and then organize these neurons/synapses into a multi-layered hierarchical structure that mimics the organization of the retina. Without using a programming language, they can then generate a simulation of this structure. Furthermore, the parameters of these equations, corresponding, for example, to physiological parameters, can be modulated via an interface. This makes it possible to vary “manually,” e.g., the conductance of an ion channel or the intensity of a synaptic connection. Thanks to this flexibility, Macular is not limited to modeling the retina alone but also allows thalamic or cortical extensions to be added. An example of a cortical extension is provided in Section 6.2. Finally, with Macular, we wanted to study the response to realistic visual stimuli, such as those used in experiments. Thus, Macular accepts films as “visual” input (this feature is inherited from Virtual Retina). However, the retina does not always receive visual input. During development, before birth, when photoreceptors are inactive, there are nevertheless electrical activities (retinal waves) that we wanted to simulate. Another situation concerns retinal prostheses, where the “input” is electrical stimulation, which is also possible with our simulator.

Macular runs on the three main operating systems: Linux, Mac, and Windows. This article provides a brief overview of the platform, noting that more comprehensive online documentation is available in the online documentation page. The article is structured as follows. In Section 2 we provide a general presentation of Macular, its spirit and structure. In Section 3 we present the GUI and its main features. In Section 4 we expose how to create cells or synapses of a new type using the Macular Template Engine. Macular also has a batch version presented in Section 5. Section 6 provides a few examples of use cases, including the simulation of retinal waves based on a model published in Cessac and Matzakou-Karvouniari (2022) and (Karvouniari et al. 2019) and a retino-cortical model published in Emonet and Cessac (Submitted)^1^ and (Emonet et al. 2025). Section 7 shortly presents existing simulators in the spirit of Macular and compares them to our platform.

## 2 General presentation

### 2.1 Installation

Macular is free software (GPL), written in C++, with the license number IDDN.FR.001.020016.001.S.P.2022.000.31235. It can be freely downloaded at this url by following the instructions given at this page. A git repository is available here.

### 2.2 Overview

The entire structure and concept of Macular rely on the following observation. The biophysics of the retina and the visual system can be modeled, with excellent accuracy, by (partial or ordinary) differential equations. These equations are, in general, complex, non-linear, with many degrees of freedom, multiple space and time scales, and have non-stationary (visual) inputs. Still, it is possible to simulate them using adapted numerical schemes and structures.

**Figure 1:**
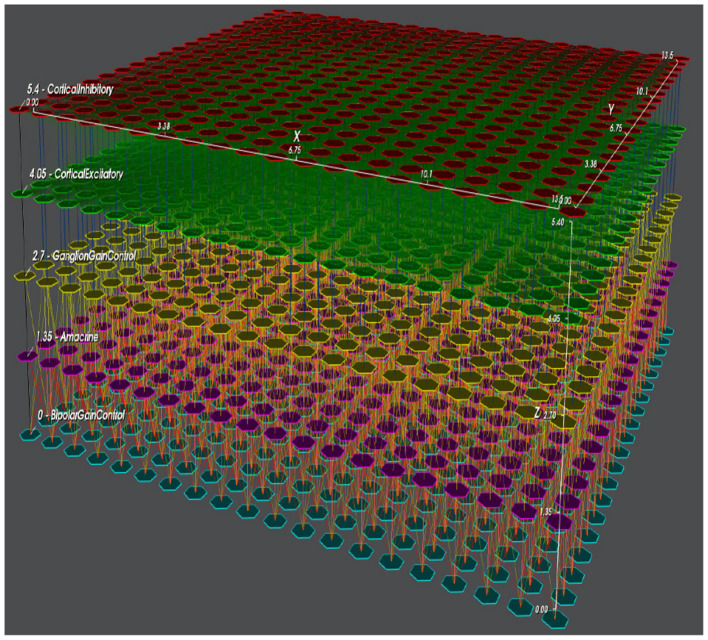
The multi-layer structure of Macular. Here, we show a scenario involving three retinal layers and two cortical layers, further described in Section 6.2. This scenario has been used in the papers (Emonet et al., 2025)^1^.

The Macular platform is organized into a layered structure that mimics the multi-layer organization of the visual system (Figure 1). It is fed by visual inputs (movies) and then processed by this multi-layer structure. At the heart of Macular are objects called “cells,” inspired by biological neurons but more general. A “cell” can also be a group of neurons of the same type, a neural field generated by a large number of neurons (for example, a cortical column), or even an electrode in a retinal prosthesis. To differentiate biological cells from Macular cells, we will use a capital in the latter case. More generally, Macular objects, such as synapses and currents, will be designed with a capital. A cell is defined by internal variables (evolving over time), internal parameters (adjusted by cursors), a dynamic evolution (described by a set of differential equations), and inputs. Inputs can come from an external visual scene or from other synaptically connected cells. Synapses are also Macular objects defined by specific variables, parameters, and equations. Cells of the same type are connected in layers according to a graph with a specific type of synapses (intra-Layer connectivity). Cells of different types can also be connected via synapses (inter-layer connectivity).

All the information concerning the types of cells, their inputs, their synapses, and the organization of the layers is stored in a.mac (for “Macular”) file, which defines what we call a “scenario.” Different scenarios are offered to the user, which they can load and play while modifying parameters and viewing variables. More generally, Macular is built around a central idea: its use and its graphical interface can evolve according to the user's objectives, so the user can design their own scenarios, i.e., define their own cells, synapses, and layers, using a specific template, the Macular Template Engine. This template, and more generally, Macular, has been designed so that the user does not need to use computer programming to run their simulations.

Although Macular targets simulations of the retina, it is not limited to it. It is designed to propose and test models of the visual system, where, for example, cells represent cortical columns in a mean-field model. However, Macular is, by no means, intended to simulate the retina or the early visual system *as a whole*. Instead, it is designed to test hypotheses about *specific* aspects of the visual system and to reproduce specific experiments *in silico*. It is a tool for modelers and experimentalists. In particular, one can present the same stimuli as experimentalists and then record the responses of cells and synapses in the model layers. This is why the notion of a user-built scenario is central. From this perspective, note that generating a model or scenario requires a clear idea of the equations to use, their parameters, and, last but not least, a coherent set of physical units. Thus, proposing a realistic scenario requires an important design phase.

### 2.3 Units

Macular uses a set of physical units listed in Table 1. There is a default system of units, shown in the second column of the table. Macular converts the user's model units to the default units for computations, then reconverts them to the user's units for plots. Notably, space scales have 3 possible “modalities”: distance, angle, or pixels (see the online documentation page for more detail). We note, however, that Macular does not check that the user's units are coherent, in contrast, e.g., to BRIAN (Goodman and Brette, 2008).

**Table 1:** Physical units used in Macular.

**Physical quantity**	**Default Macular units**	**Other possible units**
Time (τ)	Second (s)	Milli-second (ms)
Voltage (*V, E*)	Milli-volts (mV)	Volts (V)
Electric current (*I*)	Pico-ampère (pA)	Nano-ampère (nA), micro ampère per *cm*^2^ (μ/*cm*^2^)
Electric conductance (*g*)	Nano-siemens (nS)	Pico-siemens (pS), milli-siemens per *cm*^2^ (mS/*cm*^2^)
Distance (σ)	Millimeters (mm), degrees (°), pixels (px)	Micro-meters (μ*m*)
Capacitance (*C*)	Nano-farad (nF)	Pico-farad (pF), micro-farad per *cm*^2^ (μ*F*/*cm*^2^)
Frequency (ν, *f*)	Hertz (Hz)	Kilo hertz (*kHz*)
Molarity (*M*)	Nano-mol per liter (nM)	Milli-mol per liter (*mM*), micro-mol per liter (μ*M*)

The first column displays the name of the main physical quantities used in models, with the letter that usually identifies them (in parentheses). In the second column, we show the default unit of these quantities in Macular. The third column presents the other units available in Macular. In the Macular interface (GUI) "micro" is denoted u instead of μ.

### 2.4 Core architecture

We assume here that the reader knows the retina structure (for a very didactic introduction, see e.g., the web vision page by Helga Kolb). In the following lines, for simplicity with respect to the biological reality, we call *OPL* (Outer Plexiform Layer) the retina region that contains photoreceptors (rods and cones) and Horizontal cells (HCs), and *IPL* (Inner Plexiform Layer) the region that contains Bipolar cells (BCs), Amacrine cells (ACs), and Retinal Ganglion cells (RGCs). More generally, we extend the notion of layers to models containing cortical populations, each population corresponding to a layer.

#### 2.4.1 Visual flow

In Macula, the OPL is essentially represented by BCs receptive field (RF). Biologically, the RF of a BC is a region of the visual field (the physical space) in which stimulation alters its voltage (evokes a response of the cell). This definition generalizes to other retinal cell types, such as ACs or RGCs, but we stick to BCs here. In Macular, we model the receptive field of a BC *i* of type *T*, *T*_*i*_, as a spatio-temporal kernel *K*_*T*_*i*__(*x,y,t*), i.e., a function of space and time with a specific structure. This receptive field features lateral inhibition from horizontal cells in the form of a difference-of-Gaussians.

The linear response of the RF to a visual stimulus is then given by a space-time convolution (see e.g., this web page).

In Macular, convolutions are computed using a fast method called Deriche filters (Deriche, 1987) and are handled by the Virtual Retina simulator, developed by Wohrer and Kornprobst (Wohrer and Kornprobst, 2009), integrated in Macular. As a consequence, filters have a *spherical symmetry*. This limitation is further discussed in the conclusion section. Stimuli are considered as levels of gray between [1, 255]. We do not handle color in Macular. A detailed description of the Virtual Retina implementation in Macular can be found here.

#### 2.4.2 Cells

We now define Macular Cells more specifically. A cell is denoted *T*_*i*_ where *T* is called the “cell type” and *i* is the index labeling cells of type *T*. A cell type can refer to either the cell's biological classification (e.g., bipolar or amacrine retinal cell layers), to subtypes within these general cell layers (e.g., starburst amacrine cells), or to its function (e.g., ON cells). However, as already mentioned, a Macular Cell does not necessarily correspond to a biological cell. It can be, for example, a region in the cortical space (e.g., a cortical column) corresponding to a mean-field average over thousands of neurons (see Section 6.2). A glossary of (default) Cell types existing in Macular is given in Table 2.

**Table 2:** Cell types pre-defined in Macular, listed in alphabetic order.

**Cell name in Macular**	**Equation**	**Comment**
macularCellAmacrine, macularCellBipolar	$\frac{dV}{dt}=-\frac{V-E_{L}}{\tau }+V_{syn}$	Linear cell with synaptic input (*V*_*syn*_) and characteristic time τ. *E*_*L*_ is the leak reversal potential.
macularCellAmacrineLinearPharma, macularCellBipolarLinearPharma, macularCellGanglionLinearPharma	$\frac{dV}{dt}=-\frac{g_{L}+g_{P}}{C}V+\frac{I_{syn}}{C}+\frac{g_{L}E_{L}+g_{P}E_{P}}{C}$	Linear cell with membrane capacitance *C* and a tunable ion contribution e.g. corresponding to an injected drug where *g*_*P*_: conductance; *E*_*P*_: Nernst potential, of the ionic channels sensitive to that drug (see Kartsaki et al., 2024)
macularCellAmacrineGABA, macularCellAmacrineAMPA	$\frac{dT}{dt}=-k_{d}T+\frac{k_{p}}{1+e^{\left(-\left(V-E_{N}\right)/\kappa_{N}\right)}}$ $\frac{dV}{dt}=-\frac{V}{\tau_{A}}+\frac{I_{syn}}{C_{A}}$ $\frac{dn}{dt}=-\beta_{n}n+\alpha_{n}T\left(1-n\right)$	Linear Amacrine cell producing GABA (resp. AMPA) with a quasi static production of neurotransmitter T and an activation variable n. From (Destexhe et al. 1998)
macularCellBipolarGainControl	$\frac{dA_{B}}{dt}=-A_{B}/\tau_{A_{B}}+h_{B}N_{B}\left(V\right)$ $\frac{dV}{dt}=-\left(V-E_{L}\right)/\tau_{B}+Vext/\tau_{ext}+V_{syn}$	Bipolar Cell with a gain control controlled by a non linear function *N*_*B*_ of the voltage V and of an activity variable *A*_*B*_ (from Berry et al., 1999; Chen et al., 2013; Souihel and Cessac, 2019).
	The equations are too long to be written in this table. For further detail see (Zerlaut et al. 2018); (Emonet et al. 2025)	Respectively excitatory and inhibitory populations of a cortical column. Excitatory populations come from regular spiking cells (RS) and inhibitory populations from fast spiking cells (FS).
	$\frac{dV}{dt}=-\frac{V}{\tau }+\frac{I_{ext}}{C}$	Passive (low pass) electrode receiving an input, *I*_*ext*_, corresponding to a local pixel average (see Section 2.4.8).
	$\frac{dV}{dt}=-\frac{1}{\tau_{L}}\left(V-V_{L}\right)+V_{syn}-\frac{g_{T}}{C_{G}}\left(V-V_{T}\right)$; $\frac{dA_{G}}{dt}=-\frac{A_{G}}{\tau_{G}}+H_{G}N_{G}\left(V\right)$	Ganglion cells with gain control (from Berry et al., 1999; Chen et al., 2013) and a tunable ion contribution e.g., corresponding to an injected drug. Firing rate is controlled by a non linear function *N*_*G*_ of the voltage V and of an activity variable *A*_*G*_.
	From (Hodgkin and Huxley 1952).	Hodgkin-Huxley neuron with the classical form Equation 2 or with a voltage form Equation 3.
	From (Morris and Lecar 1981).	Morris-Lecar neuron.
	Used to feature Starburst Amacrine Cells. Parameters have been tuned according to the paper (Cessac and Matzakou-Karvouniari, 2022)	Morris-Lecar neuron producing Acetylcholine.
	Used to feature Starburst Amacrine Cells during development. Parameters have been tuned according to the paper (Cessac and Matzakou-Karvouniari, 2022)	Morris-Lecar neuron producing Acetylcholine with a potassium slow After Hyperpolarization current.

New Cell types can be created using the Macular Template Engine (Section). Once a variable has been introduced in the table, we do not repeat its definition. More detail can be found by clicking on the variable name in the Macular GUI.

The Cell *T*_*i*_ is identified by

- **An Input**, $\vec{I}\left(T_{i}\right)$**(*t*)**. The cell receives an entry, which can be:

1. An external Input $\vec{I}{\left(Ti\right)}_{ext}$. For example, an entry corresponding to the input from OPL (visual flow, i.e. the convolution of a movie with the OPL receptive field) to bipolar cell $\vec{I}{\left(Ti\right)}_{OPL}$ (defined in Section 2.4.1), or the electric current provided by an electrode $\vec{I}{\left(Ti\right)}_{stim}$ (defined in Section 2.4.8).
2. A synaptic input I(*Ti*)*syn*(*t*) corresponding to synaptic connections with other cells and defined in Section 2.4.5. In general, this contribution summarizes the connections with several presynaptic cells.

The input $\vec{I}\left(Ti\right)\left(t\right)$ is, in general, the sum of several contributions (e.g., OPL current and synaptic input).

- **A State**. This is an array ${\vec{X}}^{\left(T_{i}\right)}$ of *variables* evolving in time and characterizing the Cell's dynamical evolution. For example, State variables can be a membrane potential, activity—probability that an ion channel of a given type is open, concentration of neurotransmitter of a given type released by the cell, etc.
- **A set of Parameters**. These are quantities that do not change over time but are nevertheless necessary to constrain the cell's evolution. They can, for example, correspond to conductances, reversal potentials, membrane capacitance, etc. They can be modified by the user using sliders or by entering a value in a field. We denote by ${\vec{\mu }}^{\left(T_{i}\right)}$ the array of these parameters.
- **A function**, called Vector Field ${\vec{F}}^{\left(T_{i}\right)}$, controlling the time evolution of Cells. Mathematically, ${\vec{F}}^{\left(T_{i}\right)}$ is the vector field of the differential equation:

$$\begin{array}{l} \frac{d\vec{X}\left(Ti\right)}{dt}=\vec{F}\left(Ti\right)\left(\vec{X}\left(Ti\right),\vec{\mu }\left(Ti\right),\vec{I^{\left(i\right)}}\left(t\right)\right), \end{array}$$ (1)

and $\vec{F}\left(Ti\right)$ has the same dimension as $\vec{X}\left(Ti\right)$, the state vector.

#### 2.4.3 Pre-defined cell types

There is a set of pre-defined cells defined in Macular listed in Table 2. The user can create new cells using the MacularTemplateEngine presented in Section 4. Most of the predefined Cells in Macular (except the so-called “CorticalCells” which actually physically correspond to cortical columns) are based on the generic equation for voltage:


$$\begin{array}{l} C\frac{dV}{dt}=-g_{L}\;V-E_{L}\;-\underset{X}{\sum }g_{X}\;V-E_{X}\;+I_{syn}+I_{ext} \end{array}$$ (2)

where *g*_*L*_ and *E*_*L*_, respectively, refer to leak conductance and leak reversal potential, *g*_*X*_ and *E*_*X*_ correspond to ionic current contributions, *I*_*syn*_ is the synaptic current discussed in Section 2.4.5 and *I*_*ext*_ is the input current. Another form, also used in Macular, is as follows:


$$\begin{array}{l} \frac{dV}{dt}=-\frac{V-E_{L}}{\tau_{L}}-\underset{X}{\sum }\frac{V-E_{X}}{\tau_{X}}+V_{syn}+V_{ext}. \end{array}$$ (3)

It corresponds to Equation 2 setting $\tau_{L}=\frac{C}{g_{L}}$, $\tau_{X}=\frac{C}{g_{X}}$. The term *V*_*syn*_ appearing in Equation 3 corresponds to the synaptic input, explained in Section 2.4.5. Notably, it does not have the dimension of a voltage. Its dimension is *mVs*^−1^ and would correspond, from Equation 2, to $\frac{I_{syn}}{C}$. We adopted the letter *V* for simplicity. The same remark holds for the physical dimension of *V*_*ext*_. *I*_*ext*_ and *V*_*ext*_ correspond to different stages of integration in the OPL.

We distinguish 3 main cell subtypes, based on the mathematical implementation of the conductances *g*_*X*_:

- **Linear cells**. The conductances *g*_*X*_ are constant, i.e., they do not depend on any variable.
- **Rectified cells**. The conductances depend on voltage only, and take the form.
  *g*_*X*_(*V*) = λ*N*_*X*_(*V*) where λ is a constant and:

$$N_{X}(V)=\left\{ \begin{array}{l} V-\theta_{X},\text{ if }V>\theta_{X};\\ 0\text{ otherwise} \end{array}\right.,$$ (4)

is a piecewise linear rectifier, θ_*X*_ being a voltage threshold.

- **Non-Linear cells**. The conductances depend non-linearly on voltage and on potential additional variables like activation or inactivation variables. This is the case, e.g., for cells inspired by the Morris-Lecar (Morris and Lecar, 1981) or Hodgkin-Huxley models. model (Hodgkin and Huxley 1952).

In addition, some cell types have activation variables used for synaptic computation (see Section 2.4.5). Notably there is no constraint for the user to stick to cells of the form Equation 2 or Equation 3. They are free to develop their own using the Macular Template Engine (Section 4).

#### 2.4.4 Cell layers

Macular is organized into layers. A cell layer is a set of cells of the same type *T*, where “same type” means that the inputs, state vector, parameters vector, and vector field have the same mathematical expression. In this respect, Macular Layers differ from biological “layers” that can contain different cell types. Notably *Cells in the same Layer share the same set of parameters*. The State values can differ, depending on the initial conditions and on the Input. In the macula, cells are considered points; i.e., the soma, axons, and synapses of neurons are located at the same point. They are identified by an index (ID). Cells within a given layer are organized in a two-dimensional grid, and different cell layers are located in a 3-dimensional space with coordinates (*x, y, z*). All Cells of type *T* have the same *z* coordinate. Thus, the Cell *Ti* has coordinates (*x*_*i*_, *y*_*i*_, *z*_*T*_) where the vertical coordinate *z*_*T*_ parametrizes the Cell's type and the coordinates (*x*_*i*_, *y*_*i*_) the position of Cell *i* in the Layer *T*. All Layers have a common frame, with parallel axes in the *x, y* directions and a common origin. Layers are represented as rectangles. The number of cells in the horizontal and vertical directions might not be the same, and each Layer may contain a different number of cells.

#### 2.4.5 Synapses

Biological cells can be connected via chemical synapses or electric synapses (gap junctions). The synaptic contact between two cells involves complex dynamical processes such as calcium influx, release, diffusion, and capture of neurotransmitters, the opening or closing of ion channels, and the resulting electric currents that modify the membrane voltage of the post-synaptic neuron. In neuron modeling, these mechanisms are described by equations that capture different aspects of synapse dynamics. In the macula, the structures called synapses implement these aspects. A synapse is noted *Sk* where *S* is called the “synapse type” and *k* is the index labeling Synapses of type *S*. The “type” of the synapse refers here to a model, a set of equations, corresponding to a biological synapse, for example, a cholinergic synapse between two amacrine cells. The synapse connects a pre-synaptic Cell *Ti* to a post-synaptic Cell *T*′*j*.

A synapse is identified by the following:

- **A set of Parameters**. These are quantities that do not evolve in time but constrain the connectivity function of the synapse. These can be conductance, connectivity weights, or reversal potentials. They can be modified by the user using sliders or by typing a value into a field.
- **A function**, the mathematical representation of the synaptic connection. It could compute either a synaptic current current (*I*(*Ti*→*T*′*j*)*syn*), a voltage (*V*(*Ti*→*T*′*j*)*syn*) (i.e. a Post Synaptic Potential) or a firing rate (*FR*(*Ti*→*T*′*j*)*syn*). These quantities depend, in general, on the state vector of pre- and post-synaptic cells.

The predefined types of Macular synapses are listed in Table 3.

**Table 3:** Synapses type pre-defined in Macular.

**Synapse name**	**Definition**	**Comment**
macularSynapseAcetylcholine	*I*(*Tpre*→*T*′*post*)*syn*(*t*) $=-g_{A}\frac{A_{pre}^{2}}{\gamma_{A}+A_{pre}^{2}}.\left(V_{post}-V_{A}\right)$	Model of Ach conductance for nicotinic receptors (from (Karvouniari et al. 2019)). *A*_*pre*_, is the Ach concentration emitted by the pre-synaptic cell (so the Cell type must contain this variable, for example a macularCellSAC defined in table **??**); *V*_*post*_, voltage of the post-synaptic cell; *g*_*A*_, Max Ach conductance; γ_*A*_, half-activation constant; *V*_*A*_, reversal potential for Ach.
macularSynapseAmacrineToBipolar, macularSynapseAmacrineToGanglion, macularSynapseBipolarToAmacrine, macularSynapseBipolarToGanglion, macularSynapseLinearRectified	*V*(*Tpre*→*T*′*post*)*syn*(*t*) $=w_{post}^{pre}N_{pre}\left(V_{pre}-\theta_{pre}\right)$	Rectified synapse (in mV/s). $w_{post}^{pre}$, synaptic weight from pre-synaptic to post-synaptic Cells; N, linear rectifier; θ_*pre*_, rectifying threshold (mV).
macularSynapseAmacrineToBipolar, macularSynapseBipolarToAmacrine	$V\left(Tpre\to T^{\prime }post\right)syn\left(t\right)=w_{post}^{pre}V_{pre}$	Linear synapse (in mV/s).
macularSynapseBipolarGainControlToAmacrine, macularSynapseBipolarToAmacrine, macularSynapseBipolarPooling	$V\left(Tpre\to T^{\prime }post\right)syn\left(t\right)=w_{post}^{pre}preBipolarResponse$	The post synaptic voltage is proportional to the pre-synaptic voltage via a synaptic weight $w_{post}^{pre}$. The term "preBipolarResponse" depends on the Macular Cell type.
macularSynapseGapJunctionVoltage	*V*(*Tpre*→*T*′*post*)*syn*(*t*)= −*w*_*gap*_.(*V*_*post*_−*V*_*pre*_)	Passive gap junctions where *w*_*gap*_ is expressed in *nS*/*nF* = *Hz*.
macularSynapseCorticalExc_to_CorticalExc, macularSynapseCorticalExc_to_CorticalInh, macularSynapseCorticalInh_to_CorticalExc, macularSynapseCorticalInh_to_CorticalInh	$\nu_{post}=w_{post}^{pre}.\nu_{pre}$	From (Zerlaut et al. 2018), where ν_*pre*_, ν_*post*_ are the firing rates of the pre/post-synaptic Cell (corresponding here to a cortical column) and $w_{post}^{pre}$ the gaussian weigth between pre/post-synaptic Cell.
macularSynapseGABA_A, macularSynapseAMPA	$V\left(Tpre\to T^{\prime }post\right)syn\left(t\right)=-gn\left(V_{pre}-E\right)$	Here *n* is an activation variable as produced by the Cells macularCellAmacrineGABA, macu-larCellAmacrineAMPA.
macularSynapseRetinoCortical	$FR_{syn}^{T_{pre}\to T_{post}^{\prime }}\left(t\right)=weight.\frac{density_{retine}}{density_{cortex}}.preFiringRate$	Synapse connecting the retina to the cortex (Souihel 2019) where preFiringRate is the output firing rate of ganglion cells. It is multiplied by a factor corresponding to the ratio between the retinal density and the cortical density. Respectively 400*mm*^−2^ and 4000*mm*^−2^ in the model (Emonet et al., 2025).

New Synapses type can be created using the Macular Template Engine (Section 4).

In the Macular Graph Generator (see Section 3.2.2), the user specifies the cell type in each layer and selects the synapse type within a layer (intra-Layer synapses) or between Layers (inter-Layer synapses). There can be several types of intra- or inter-layer synapses in the simulation.

A post-synaptic cell receives, in general, many inputs from different cells of different types. Thus, the general form of the Synaptic Current I(*Ti*)*syn*(*t*) introduced in Section 2.4.2, Equation 2, is:


$$I_{syn}\equiv \;I_{syn}^{\left(T_{i}\right)}(t)=\sum_{T^{\prime }}\sum_{j\in T^{\prime }}\;I_{syn}^{\left(T_{i}\to T_{j}^{'}\right)}(t),$$ (5)

where the first summation holds on the cells *j* of type *T* pre-synaptic to cell *i*, and the second summation holds on cells layers. The same formulation holds for the voltage representation introduced in Section 2.4.2, Equation 3:


$$V_{syn}\equiv V_{syn}^{T_{i}}(t)=\sum_{T^{\prime }}V_{syn}^{\left(T_{i}\to T_{j}^{'}\right)}(t),$$ (6)

or a firing rate input used, e.g., for retino-cortical Synapses:


$$FR_{syn}^{T_{i}}(t)=\sum_{T^{\prime }}\sum_{j\in T^{\prime }}FR_{syn}^{\left(T_{i}\to T_{j}^{'}\right)}(t),$$ (7)

By default, synapses in Macular are instantaneous, i.e., there is no delay between the emission of a signal at the presynaptic neuron and its arrival at the post-synaptic neuron. It is nevertheless possible to add a delay to a Synapse type. For this, the user must create a speed parameter named “conduction_velocity” in the synapse type. Macular computes the synaptic delay using the equation:


$$\begin{array}{l} delay_{syn}=\frac{d_{syn}}{v_{C}}, \end{array}$$ (8)

where *d*_*syn*_ is the distance between the two neurons (e.g., the length of the axons), and *v*_*C*_ is the conduction velocity.

#### 2.4.6 Graph

Synapses define a natural notion of intra- and inter-layer connectivity. If the cell *Ti* is pre-synaptic to Cell *T*′*j*, with a Synapse of type *S*, we note $Ti\overset{S}{\to }T^{\prime }j$ the oriented edge featuring this connection. The set of edges of type *S* defines a directed graph $G^{\left(T\overset{S}{\to }T^{\prime }\right)}$. This graph features the set of synaptic connections of type *S*, from Layer *T* to Layer *T*′. If *T* = *T*′, we speak of “intra-Layer connectivity” of type *S*, and “inter-Layer” if *T*≠*T*′. Between two Layers, there may be several types of synaptic connections, and a cell can be a source or target for different types of Synapses (e.g., an AC can connect a BC through a glycinergic synapse and a gap junction).

In this frame, Cell *i* has coordinates *x*_*i*_, *y*_*i*_ in its Layer, while Cell *j* has coordinates *x*_*j*_, *y*_*j*_ in its Layer. The distance between these two cells is $d\left(i,j\right)=\sqrt{{\;x_{i}-x_{j}\;}^{2}+\;y_{i}-y_{j}^{2}\;}$, the two-dimensional Euclidean distance. That is, we do not consider the vertical distance between different Layers. Two cells are nearest neighbors if their distance is the smallest strictly positive distance.

In Macular, there are six types of connectivity that a graph can implement between two Layers:

- **One-to-one (Inter-Layers)**. A cell is connected to the cell at zero distance in another Layer. This type of connectivity requires that these Layers have the same number of cells.
- **Nearest neighbors (Inter- and Intra-Layers)**. A cell is connected to its four nearest neighbors.
- **Neighbor 4 + 1 (Inter-Layers)**. A cell is connected to 4 nearest neighbors and to the cell at distance zero.
- **Radius neighbors (Inter- and Intra-Layers)**. A cell is connected to neighboring cells within a certain radius (excluding the cell at distance zero). The synaptic weights are constant within this radius.
- **Gaussian (Inter- and Intra-Layers)**. The synaptic weight between the pre-synaptic and the post-synaptic cell depends on their distance *d*(*i, j*), via a Gaussian profile:

$$W_{post}^{pre}=\frac{e^{-\frac{d{\left(i,j\right)}^{2}}{2\;\sigma_{p}^{2}}}}{2\pi \sigma_{p}^{2}}$$ (9)

In this case, we have connectivity to the cell at a distance of 0.

- **Fully Connected (Inter-Layers)**. A cell is connected to all cells with constant synaptic weights.

Among these connectivity types, Gaussian and Nearest Neighbors are currently the only ones used to connect cells within the same Layer.

#### 2.4.7 ODE solver

Macular integrates ordinary differential equations (ODE) using the General Scientific Library (GSL) (See the GSL online documentation). The library provides a variety of low-level methods, such as Runge-Kutta and Bulirsch-Stoer routines, as well as higher-level components for adaptive step-size control. By default, Macular uses the Runge-Kutta of order 4 (RK4) method. Note therefore that the current implementation of Macular is not adapted to simulate evolution with noise (which would require specific stochastic integrators). The Macular GUI menu allows the selection of different integration methods: RK2, RK4, RK45, RK8, RKCK, RK1imp, RK2imp, RK4imp, BSIMP, ADAMS, BDF. See the online documentation of the GSL for details on these methods.

#### 2.4.8 Electrodes stimulation

Retinal implants are electronic devices surgically attached to the retina. They replace defective cells to partially restore vision. Images acquired by a “camera + processor” system are encoded and sent as pulses to a matrix of electrodes. It then stimulates the still-functioning cells of the retina to produce a luminous impression.

We have implemented a simplified version of this process on Macular. Electrodes are considered as “cells” (type macularCellElectrode). They are quite simplified compared to real electrodes, as they are just low-pass filters, but the user can extend their definition using more complex equations and the MacularTemplateEngine facilities (Section 4). A retinal prosthesis is a matrix of electrodes that, in the macula, becomes a matrix of “macularCellElectrode”.

The “camera + processor” processing is featured by averaging the pixels around the location of a given macularCellElectrode in a region whose size is the image size in pixels divided by the number of electrodes. This averaging provides the macularCellElectrode input. This functionality is obtained by selecting “Prosthesis” in the “WorkerSetting” (see Section 3.2).

## 3 The Macular GUI

Macular has a Graphical User Interface (GUI) with a large set of options, such as visualizing the cell Layers in 2D or 3D, and monitoring specific cell State variables … . The majority of the elements in the Macular GUI have small embedded documentation that appears when the mouse is pointed at them.

### 3.1 Views

When opening Macular, a panel appears showing up 4 buttons corresponding to different views.

- **3D view** creates a “canvas” object that provides a layered, customizable view of the simulation.
- **Layered view** provides a set of 2D views “Views2D”, one for each Layer, and is customizable.
- **Plot views** allows the generation of a Plot2D object to monitor the time evolution of specific cells variables.
- **Stimulus**. When an image or a video is played, this option allows one to see the stimulus.

Several views can be simultaneously open.

### 3.2 Simulator

#### 3.2.1 The configuration panel

On the left of the GUI a list of icons is visible. This is the configuration panel, respectively corresponding to the following functions.

- **Selection**. The user can select which output they want to record in their simulation.
- **Video Input**. The command **Browse Stimulus** loads a visual stimulus in the form of a movie in the formats .mp4, .mkv, .avi. This stimulus will be played when running the simulation.
- **Graph Input**. The command **Browse Graph** loads a .mac (mac, for “Macular”) file containing a Macular graph (see Section 3.2.2 for a description of the .mac files).
- **Worker settings**. **Input** selects a Worker, namely a setting of functionalities to run the simulation according to the type of visual input. The options are:

1. “Visual Flow”. The menu essentially contains parameters that shape the Receptive Field filter (see Section 2.4.1).
2. “Prosthesis”. Here, one parametrizes the setting for retinal prostheses.
3. “None”. Here, there is no input.

- **Simulation parameters**. This menu allows to further parameterize the simulation.
- **Controls** allow the simulation to be run, saved, and reset.
- **Parameters of cells and Synapses**. Here, one can select a predefined cell or Synapse type.
- **Configuration** allows the user to select the appearance of the GUI (colors of the background, fonts) and to toggle advanced parameters selected in the parameters visibility view.

#### 3.2.2 The graph generator

The Graph Generator allows the user to create layers of cells with a given connectivity using the existing cell/Synapse types (for creating new cell types, see Section 4). An example of Graph creation is provided here. As exposed in Section 2.4.6, a Graph is a mathematical structure made up of vertices ( cells), connected by intra- and inter-layer edges (Synapses). In Macular, a graph is implemented as a C++ object. The data necessary to run the simulation are saved in two files. The first one, with the extension .mac (mac, for Macular), contains the number of cells, the number of Synapses, the type of each Cell with its coordinates, the type of each Synapse, and the cells it connects to, and, finally, the initial value of each variable. The second file, with the.json extension, contains information about the model parameters. More details can be found in the online documentation page.

## 4 The Macular Template Engine

The Macular Template Engine (MTE) allows the user to manage existing cell and Synapse types, to create new cell and Synapse types, or to suppress them. Then, MTE automatically generates (i.e., without writing code) a set of C++ files. After any change to MTE, the user must press “Write C++ files” and recompile Macular using the “Build” button.

**Important notice**. Modifying existing cells or Synapses will replace the users existing files, except for protected cell and Synapse types. Indeed, some Macular cell or Synapse types are protected: they end with a “.lock” extension in their JSON filename. They cannot be modified by the MTE. Currently, only two Macular cells are protected (macularCellCorticalExcitatory and macularCellCorticalInhibitory).

In more detail, the main features of the MTE are:

- **Loading** the cells and Synapse types already existing in the directory share/macular/app/macularTemplateEngine/json/ (on Windows, the share directory is a subdirectory of the *Library* directory).
- **Creating and editing new Types**. This allows the user to create new cells/synapse types or to edit unprotected cell/synapse types with specific parameters, auxiliary functions, and vector field equations.
- **Deleting existing Types**. This allows the user to suppress any unprotected cell and Synapse type. The corresponding .json file (inside share/macular/app/macularTemplateEngine/json/ subfolder ) is suppressed.
- **Write C++** **Files**: With this functionality, once the new cells/Synapses types are saved in .json Files, the MTE will generate new C++/CMake files with the parameters, functions, and equations specified in the .json files. For this purpose, Macular uses Python scripts to create files from C++ templates. This script fills in the required data in the C++ templates from the .json files for each cell and Synapse. The user can finally recompile Macular in order to add these new cell/Synapse types to the Simulator and the Graph Generator. Notably this operation is performed by simply pressing the “Build” button. The source files for the generated cells and Synapses are located in /macular/share/macular/src/macularCore/, assuming that Macular is installed in /macular.

The MTE is run by typing the shell command bin/macularTemplateEngine & in the main directory (Linux) (on MacOS, one runs the file macular.app in bin, and on Windows, the binary should be available on your desktop after installation). An example of usage can be found here.

## 5 Macular batch

All features available in the Macular GUI are also available in the batch version of Macular. This version is run from the main directory by typing “./bin/macular-batch -f path_session_file.json” in a shell/terminal (on Windows, it's in Library/bin/macular-batch). The -f or - file argument is the only one mandatory. It requires the path to a Macular session JSON file. There are other options described in the the online documentation page.

## 6 Examples

Here, we provide a few examples of simulations that include scenarios from the Macular release. For more details, see the online documentation page.

### 6.1 Retinal waves

This scenario, available in the directory

**Figure 2:**
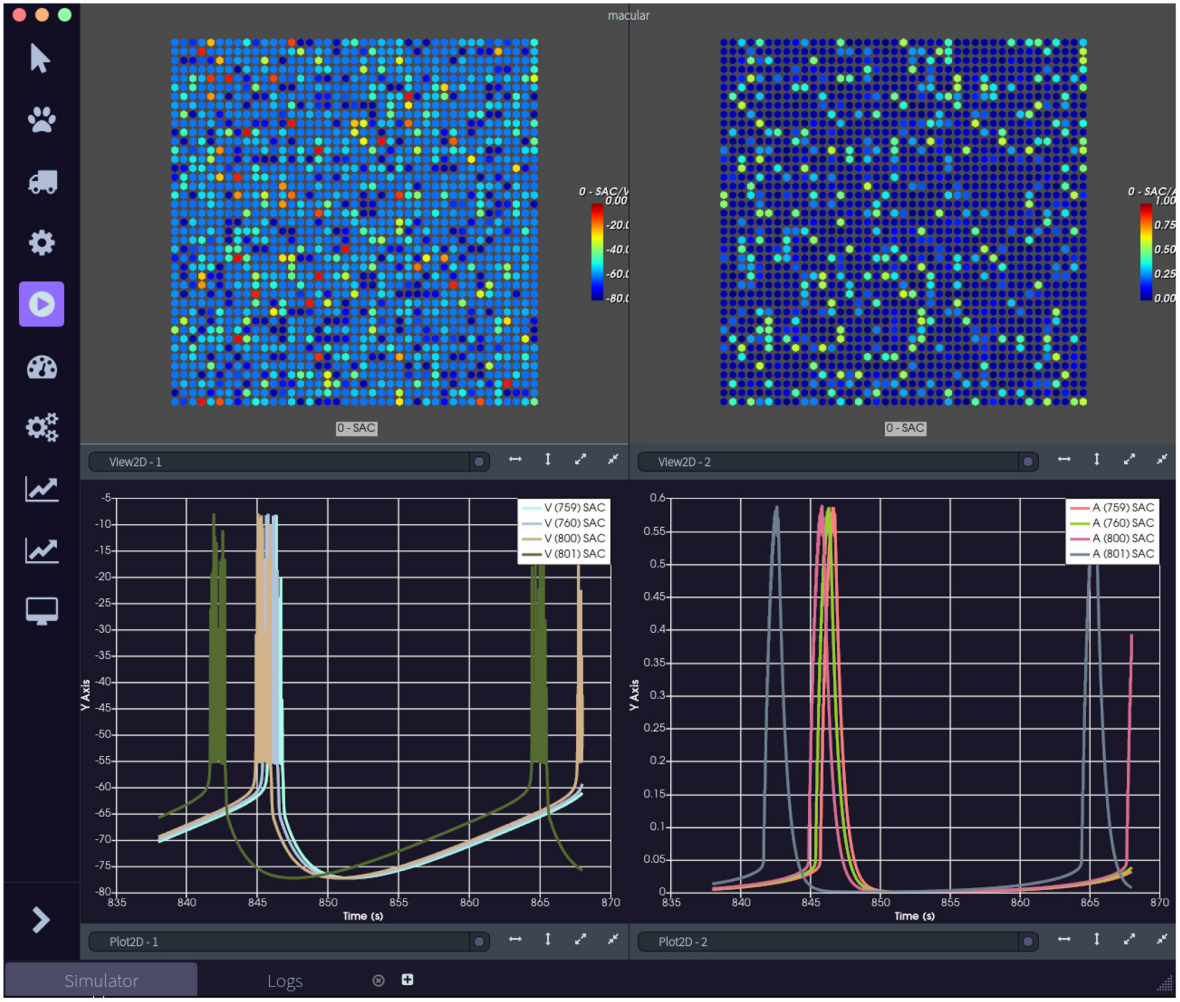
The retinal waves scenario. **(Top left)** Visualization of the lattice evolution for the voltage of Starburst Amacrine cells (SACs). **(Top right)** Visualization of the lattice evolution for the acetylcholine concentration produced by SACs. **(Bottom left)** Time evolution for the voltage of a few SACs selected by the user. **(Bottom right)** Time evolution for the acetylcholine production of a few SACs.

*macular/examples/Scenario1_RetinalWaves* (on Windows, it is located in *examples*, at the top of the Macular installation directory) provides a simulation of retinal waves occurring during the development of the visual system. It involves Starburst Amacrine cells (SAC), which are sporadically synchronizing, producing waves of bursting activity (see Karvouniari et al., 2019; Cessac and Matzakou-Karvouniari, 2022 for more detail on the model and references therein about developmental retinal waves). On this page, we detail how to create the graph and how to run the simulation and visualize the results. A view of this simulation is shown in Figure 2.

### 6.2 A retino-cortical model

**Figure 3:**
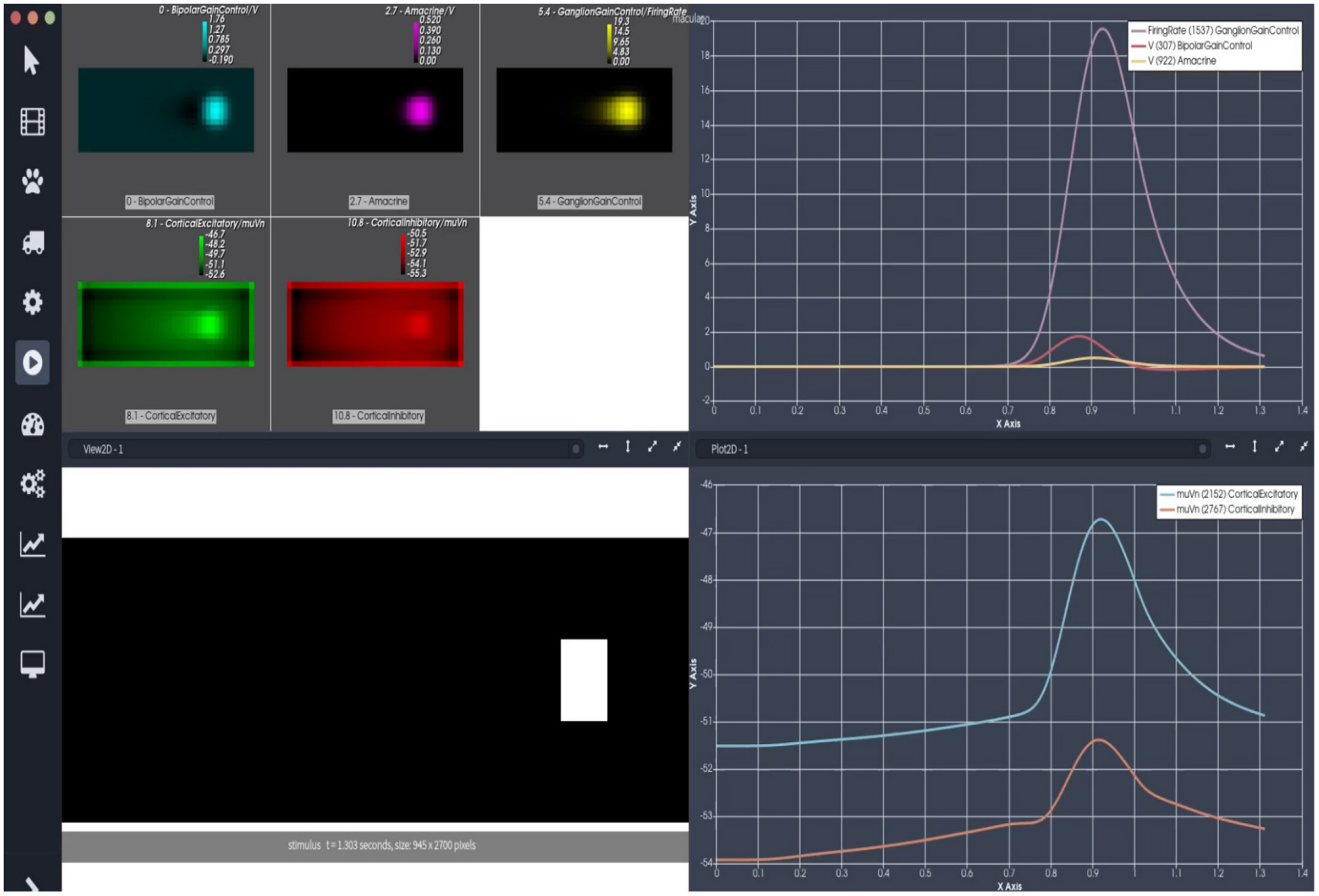
The retino-cortical scenario. The upper left panel shows the heatmap of the 5 cell types. Bipolar cells with gain control appear in blue, amacrine cells in magenta, ganglion cells with gain control in yellow, the excitatory population of cortical columns in green, and the inhibitory population of cortical columns in red. On the panel below, left, one sees the video of the stimulus, a white bar moving. The right panels are plots of cell activity. The upper one displays retinal outputs: bipolar voltage (red), amacrine voltage (yellow), and ganglion cells firing rate (pink). The bottom panel displays cortical output: excitatory (blue) and inhibitory (orange) mean voltages.

In this second example, we consider a model of retino-cortical associations that features the joint evolution of the retina and V1 in response to visual stimuli. A view of the corresponding Macular simulation is shown in Figures 1, 3.

The retina model is composed of 3 layers: bipolar cells with gain control (BCs) receiving an input from the OPL, amacrine cells (ACs) providing lateral inhibition, and retinal ganglion cells (RGCs) receiving excitation from BCs and inhibition from ACs (Souihel and Cessac, 2021; Cessac, 2022; Kartsaki et al., 2024; Emonet et al., 2025). Moreover, BCs and ACs are mutually connected. BCs excite ACs, and ACs inhibit BCs. It is possible to enable or disable the model's features (lateral connectivity and gain control) by adjusting their parameters. The cortical model of V1 features the joint evolution of two populations of cortical columns, one excitatory, the other inhibitory, coupled via delayed lateral connectivity that depends on conduction velocity, and evolving via dynamic mean-field equations with input from the retina/thalamus. This model has been proposed in (ElBoustani and Destexhe 2009), (Benvenuti et al. 2015, 2020), and (Zerlaut et al. 2018). We refer to these papers for the details. This model and this Macular scenario have been used in the papers (Emonet et al., 2025)^1^. The firing rates of RGCs constitute the inputs to the cortical model. Thus, there is no thalamus in this example.

How to create the corresponding graph and generate the simulation is described in detail there. Here, we just show the result of a simulation obtained by loading a scenario available with the Macular release. In this scenario, illustrated by Figure 3, the only feature activated is the lateral connectivity between bipolar and amacrine cells. The corresponding Macular session and graph have been placed in the “macular/examples” repository: “Scenario2_RetinoCortical.json” for the graph and “Scenario2_RetinoCortical_layout.json” for the session.

### 6.3 Creating a new model

Here, we give an example of a new cell type and Synapses created with the MTE. The detailed procedure can be found here. This example corresponds to the Amari-Wilson-Cowan model (Amari, 1971; Wilson and Cowan, 1973) whose equations reads:


$$\begin{array}{l} \frac{dV_{i}}{dt}=-\frac{V_{i}}{\tau }+\overset{N}{\underset{j=1}{\sum }}J_{ij}f\left(V_{j}\right)+H_{{ext}_{i}}\left(t\right),\;j=1\ldots N, \end{array}$$ (10)

where *V*_*i*_ is the voltage of Cell *i* in mV, τ a characteristic integration time, *J*_*ij*_ a synaptic weight (in mV/s), *H*_*ext*_*i*__(*t*) the OPL input (it has the dimension *mVs*^−1^). The function *f* is a sigmoid function of the form:


$$f(x)=\frac{1}{2}\left[1\;+\;erf\left(\frac{gx}{\sqrt{2}}\right)\right],$$ (11)

*g* being a positive parameter called “sigmoid gain” (in *mV*^−1^). This model has been widely studied in the literature, especially in the case where the *J*_*ij*_ are random independent variables, with no external current. In this case, dynamics is chaotic for a sufficiently high gain *g* (Sompolinsky et al., 1988; Cessac, 2019). Here, we consider the case where the *J*_*ij*_'s correspond to Gaussian pooling (see Section 2.4.6, Equation 9) with excitatory and inhibitory synapses and with an OPL input. This does not really correspond to a realistic situation, as Equation 10 would correspond to ganglion cells, the only spiking retinal cells, that would receive a direct OPL input. This does not hold in the real retina.

## 7 Comparison to other simulation software

Table 4 shows a comparison between different retina simulation software. We have been focusing here on software specialized for the retina. Thus, we omitted more generalist software such as COMSOL (which also has a graphical interface), MATLAB, or NEURON.

**Table 4:** Comparison between a selection of existing software and Macular.

**Intentionally missing**	**CR**	**RS**	**RT**	**CN**	**IS**	**P2P**	**RSt**	**VR**	**MA**
OS	Linux	All	Linux-Mac	Linux	All	All	x	All	All
Version	x	x	1.7.57	0.6.4	x	0.10.0	x	2.2.3	1.5.2
Language	C++	Matlab	C++	Python	Matlab	Python	Flowlang	C++	C++
Type	Library	Toolbox	Library	Toolbox	Toolbox	Library	Library	Standalone	Standalone
Dependences		Matlab		PyTorch	Matlab		x		
Open source	•	*	•	•	•	•	•	•	•
P.K.R.	•		•	•		•	•	•	
GUI									•
Videos as inputs	•			•	•	•	•	•	•
OPL modeling	S.T.K.	D.E.	x	S.T.K.	N.T.	S.T.K.	S.T.K.	S.T.K.	S.T.K.
Shape of RF	x	x	x	Any	x	N.A.	x	Circular	Circular
3D visualization									•
Scripting Interface							•		•
Parameters tuning									Sliders
Extended cells									•
Thalamus, Cortex				•	•				•
Cells recording									•
I.W.O.L.	NEST	Matlab			Matlab				
S.P.U.									•
Prostheses						•	•		•
C.I.M.									•

Abbreviations for software names have been chosen for the presentation. The following software is discussed: **CR**, COREM (Mart́ınez-Cañada et al., 2016), **RS**, “Retina Simulator” (Baek et al., 2020), **RT**, RetSim. **CN**, CONVIS (Huth et al., 2018), **IS**, ISETBio (Cottaris et al., 2019), **P2P**, Pulse2Percept (Michael Beyeler et al., 2017), **RSt**, RetinaStudio, **VR**, Virtual Retina (Wohrer and Kornprobst, 2009), and **MA**, Macular. Notably, the features selected in this table have essentially been chosen based on what Macular does. It is not an exhaustive list, and the information applies to the time of writing. The software we mention can have additional features not mentioned herein. “All” in the row “OS (operating system)” means Linux, Mac, and Windows. Other abbreviations used in this table are: D.E, Differential Equations. P.K.R, Programming Knowledge Required. I.W.O.L, Interface With Other Languages. S.P.U, Select Physical Units. C.I.M, Choice of Integration Methods. S.T.K, Spatio-temporal Kernels. N.T, Nonlinear Transformations. A • means “yes.” A white cell means “no.” A x in a column means that we haven't been able to find the information. “Extended cells,” signifies the Cell concept of Macular. * means that the referred Git page is not accessible.

## 8 Discussion

Macular was designed to maximize its accessibility, usability, and development. That is why it is distributed as free software. It is also why we have designed an interface that allows non-programmers to use it, enabling them not only to simulate existing scenarios but also to design new ones by creating new types of cells and synapses.

We would now like Macular to evolve freely, according to the communities that might use it. With this in mind, there could be several useful developments:

- **Multiple cell classes**. Macular allows for the simultaneous simulation of different cell classes (e.g., BCs, ACs, RGCs) and different types within each class. Thus, we can simultaneously simulate ON and OFF BCs. However, the input to BCs comes from VR that emulates the OPL, and that response can be either ON or OFF. We are currently working on an extended worker allowing for feature ON and OFF OPL responses simultaneously.
- **Point neurons**. In the current release, multi-compartment models of neurons are not allowed. It is, however, possible to upgrade Macular to have spatially extended neurons, although this is not planned by our group.
- **Interfaces with other simulators**. As we have shown, Macular can be used to produce a model of the V1 cortex receiving realistic retinal input (see Emonet et al., 2025). To our knowledge, this is the only example of its kind. It would be interesting to extend the integration to other cortical areas by interfacing Macular with other simulators such as TheVirtualBrain (Sanz Leon et al., 2013) or NEST (Gewaltig et al., 2001).
- **Optimization of computation time**. It would be beneficial to port Macular to parallel architectures or GPUs, which would allow for larger-scale or real-time simulations [in the spirit of (Baek et al., 2020)].
- **Generalized receptive fields**. The method we use to calculate the convolution of receptive fields with stimuli, imported from VirtualRetina, does not allow for asymmetric kernels, for example, sensitive to direction or orientation, unlike simulators such as Convis (Huth et al., 2018). It would be useful to extend the kernels of the receptive fields to a more general form, at the cost of slower calculations (Huth et al., 2018).

We hope that Macular and its extensions will enable a new type of simulation in neuroscience, allowing for integrated models of the visual system with realistic sensory inputs, i.e., dynamic and multi-scale in space and time, e.g., films presenting visual scenes from the external world.

## Data Availability

The original contributions presented in the study are included in the article/supplementary material, further inquiries can be directed to the corresponding author.

## Footnotes

1 Emonet, J., and Cessac, B. (Submitted). The refresh rate of overhead projectors may affect the perception of fast moving objects: a modelling study.
